# Application of Two-Compartment Bipolar Membrane Electrodialysis for Treatment of Waste Na_2_SO_4_ Solution

**DOI:** 10.3390/membranes15100312

**Published:** 2025-10-14

**Authors:** Young-Jae Lee, Min-Hyuk Seo, Jae-Hyuk Chang, Jun-Hee Kim, Jae-Woo Ahn

**Affiliations:** Department of Advanced Materials Science and Engineering, Daejin University, 1007 Hoguk-ro, Pocheon-si 11159, Republic of Korea; ab224009@daejin.ac.kr (Y.-J.L.); ab224008@daejin.ac.kr (M.-H.S.); ab225026@daejin.ac.kr (J.-H.C.); ab225025@daejin.ac.kr (J.-H.K.)

**Keywords:** two-compartment, bipolar membrane electrodialysis, NaOH recovery, Na_2_SO_4_ solution

## Abstract

This study evaluated the performance of a constant-current two-compartment bipolar membrane electrodialysis (BMED) system comprising cation exchange membranes and bipolar membranes for the recovery of sodium hydroxide (NaOH) from sodium sulfate (Na_2_SO_4_) solution. Key operating parameters, current density, feed concentration, initial base concentration, and solution volume, were systematically varied to investigate their effects on ion transport, NaOH concentration, current efficiency, and energy consumption. At 450 A/m^2^ with 1.30 M Na_2_SO_4_, 0.10 M initial NaOH, and 1.00 L solution volume, the system achieved a NaOH recovery yield of 69.21%, a final concentration of 2.13 M, a current efficiency of 36.39%, and an energy consumption of 1.82 kWh/kg Na_2_SO_4_ processed, corresponding to 4.72 kWh/kg NaOH produced, indicating optimal energy efficiency and process stability. To maximize concentration, the highest NaOH concentration of 2.85 M was obtained at the same current density by reducing the initial NaOH volume to 0.50 L, although this led to increased water transport and higher energy consumption (2.31 kWh/kg Na_2_SO_4_; 5.99 kWh/kg NaOH), compromising process efficiency.

## 1. Introduction

In recent years, various policies have been implemented worldwide to address environmental issues and the energy crisis, aiming to achieve carbon neutrality and realize the United Nation’s Sustainable Development Goals [1]. A major endeavor has been the development of electric vehicles (EVs), an industry that is now rapidly growing. The core components of EVs are lithium-ion batteries, which contain high-value metals such as lithium (Li), cobalt (Co), nickel (Ni), and manganese (Mn) that need to be recovered. Consequently, the related metal recycling industry is now also growing alongside the EV industry [2,3].

Generally, valuable metals such as lithium, nickel, cobalt, and manganese are recovered from used lithium-ion batteries via wet leaching technology [4]. The black mass obtained through crushing and grinding is treated via leaching and precipitation to remove impurities, followed by solvent extraction to separate metals, which are then crystallized to recover the desired metal forms. During this process, sulfuric acid and caustic soda are used to adjust the pH, resulting in the generation of large amounts of sodium sulfate (Na_2_SO_4_) as a byproduct [5,6]. Currently, Na_2_SO_4_ wastewater is commonly treated by adding calcium hydroxide (Ca(OH)_2_) and either precipitating it into calcium sulfate or evaporating and concentrating it to crystallize and recover high-purity sodium sulfate, which is then partially sold. However, both procedures have drawbacks: the chemical precipitation method, while relatively simple, produces sludge as an additional byproduct, whereas the evaporation concentration method, though capable of recovering high-purity Na_2_SO_4_, has high energy costs due to the high temperatures required [7,8]. To address these issues, electrodialysis (ED) and bipolar-membrane electrodialysis (BMED) using ion exchange membranes are being actively researched [9]. ED is an environmentally friendly process that uses only electrical energy to decompose water and simultaneously produce acids and bases. In particular, BMED has the advantage of decomposing water at a lower voltage of 0.83 V within the bipolar membrane, thus resulting in relatively low energy consumption [10].

Previous BMED-related research has primarily focused on a three-chamber structure that uses cation exchange membranes (CEMs), anion exchange membranes, and bipolar membranes (BPMs) to simultaneously recover acids and bases. This three-phase structure consists of a base recovery chamber, an acid recovery chamber, and a feed solution chamber, allowing for clear separation of the pathways of ion movement. This enables the independent production of high-purity acids and bases, minimizing cross-contamination and offering advantages in terms of product quality. Additionally, the process demonstrates flexibility in accommodating various wastewater compositions [11,12]. Nevertheless, the three-chamber structure has several limitations, including high equipment costs and operational energy consumption due to the complex stack and device configuration resulting from the large number of membranes involved. Additionally, the independent existence of a desalination zone leads to relatively high electrical resistance within the system, causing a significant drop in voltage and consequently a reduced current efficiency [13]. To address the drawbacks of the three-phase structure, a two-compartment BMED system using either a CEM or an anion exchange membrane between BPMs has recently been proposed. The two-compartment method consists of two zones: a base or acid recovery chamber and a feed solution chamber. This system has fewer membranes and a simpler structure compared to the three-chamber system, resulting in reduced equipment costs and lower electrical resistance, which in turn reduces energy consumption [14,15]. However, in the two- compartment method, while one acid or base can be recovered at high levels of purity, the other is recovered as mixed with the initial salt. Methods for acid or base recovery from the mixed salt solution require further investigation.

The two- compartment system described above is used in various fields, including organic acid recovery, Na_2_SO_4_-based wastewater treatment, and biofermentation processes. In a previous study by Xu et al., experiments were conducted to recover citric acid from Na_3_Cit using a two-compartment BMED system equipped with a CEM and a BPM. The results showed that the system recovered approximately 30 g/L of citric acid under Na_3_Cit solution at 0.1 M, a current density of 100 mA/cm^2^ and an operating time of 200 min [16]. Another study by Paleologou et al. also used a two-compartment BMED system to recover caustic soda and sulfuric acid (H_2_SO_4_) from Na_2_SO_4_, achieving a current efficiency of 78% at a current density of 142.9 mA/cm^2^ using a mixture of 1.4 M Na_2_SO_4_ + 0.25 M NaHSO_4_ solution [17]. A final example is the research by Sun et al., where a two- compartment BMED system was applied to recover citric acid from biofermentation broth, achieving a maximum recovery rate of 97.1% at a current density of 40 mA/cm^2^ using a 3.3 wt.% Na_3_Cit solution [18]. An experiment has also been conducted using a two-electrolyte BMED system including CEMs and BPMs to recover NaOH from spent Na_2_SO_4_ solution under constant voltage conditions [19]. In that research, applied voltage at 20 V, a feed solution concentration of 1.30 M Na_2_SO_4_, and an initial base concentration and volume of 0.1 M NaOH and 0.5 L, respectively, were shown to achieve an optimal NaOH recovery yield of approximately 67.6% with a high concentration of 2.96 M. However, the constant voltage method exhibits nonlinear current fluctuations due to changes in ion concentration during experiments, making precise control of ion transport difficult and limiting the evaluation of current efficiency and energy consumption. Additionally, excessive current increases and membrane damage due to increasing voltage must be considered as potential issues. Furthermore, long-term stability and membrane degradation remain critical challenges for practical BMED operation. In particular, cation-exchange membranes are susceptible to proton attack and radical-induced degradation under strongly acidic environments, which may lead to structural defects and loss of ionic conductivity [20]. The present study aimed to investigate the advantages and practical feasibility of using the same two- compartment BMED system, but operated under constant-current conditions, by quantitatively evaluating ion transfer characteristics, current efficiency, NaOH recovery concentration, and energy consumption over time.

## 2. Materials and Methods

### 2.1. Experimental Setup

**Figure 1:**
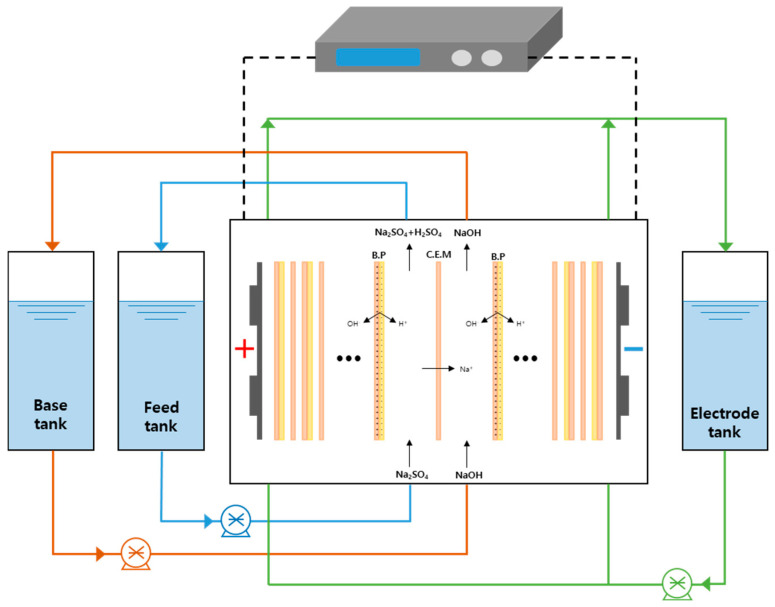
Schematic of the bipolar membrane electrodialysis system used in this study.

**Figure 2:**
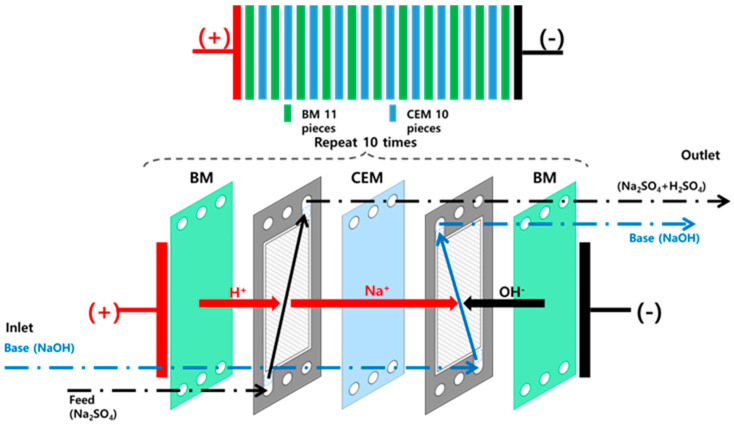
Schematic diagram of Bipolar Membrane stack.

A two-compartment membrane stack consisting of CEMs and a BPMs was installed in a BMED device manufactured in-house in Korea to perform a NaOH recovery experiment. The configuration of the device is shown in Figure 1. The system consists of a base chamber, a feed chamber, and an electrode chamber equipped with the membrane, along with auxiliary components such as a pH meter, conductivity meter, pump, and rectifier. Experimental conditions were set by adjusting the voltage and current through the control screen on the front of the device. The pH meter was installed in the base recovery zone to confirm base generation by assessing pH changes, whereas the electrical conductivity meter was installed in the feed chamber to monitor variation in conductivity in real-time during the experiment. The main specifications of the device are summarized in Table 1. The two-compartment membrane stack used consisted of 11 BPMs and 10 CEMs from the Neosepta series produced by the Japanese company ASTOM. In the stack, the two membrane types were arranged alternately. The anode and cathode were each positioned at the opposite ends, and the effective membrane area was 0.055 m^2^. Platinum-coated titanium electrodes (5 cm × 10 cm) were installed in the electrode compartments, and each recovery compartment was circulated at a flow rate of approximately 950 mL/min. Since gas evolution from water electrolysis (H_2_ at the cathode and O_2_ at the anode) occurred only in the electrode compartments, no dedicated gas removal devices were employed. The electrode compartments were separated from the adjacent feed and base recovery chambers by gaskets and spacers, and the detailed configuration is shown in Figure 2. In addition, the key physical and electrochemical properties of the BPMs and CEMs employed are summarized in Table 2. All experiments were conducted in a temperature-controlled laboratory environment at 30 ± 1 °C. To maintain stable temperature conditions during operation, an air cooling system with fans was applied. The temperatures of the feed, base, and electrode solutions were continuously monitored and recorded via the display screen of the device.

### 2.2. Experimental Methods

The feed solution used in the experiment was a simulated solution with a composition similar to that of the solution obtained by primary crystallization of Na_2_SO_4_ from wastewater generated during the recycling of lithium-ion batteries, followed by redissolution. The simulated solution was prepared and used at concentrations ranging from 0.22 to 1.30 M depending on experimental conditions. The base chamber was filled with 0.1 M NaOH solution, and the initial filling concentration and volume were varied between 0.05 and 1.00 M and between 0.5 and 1.5 L, respectively, depending on experimental conditions. A 5.0 wt.% Na_2_SO_4_ solution was used as the electrode solution. The experiment was conducted in a constant-current mode, and samples were collected at regular intervals to confirm whether NaOH was generated in the base recovery chamber and H_2_SO_4_ was generated in the raw material solution chamber over time. The collected base samples were subjected to measurement of OH^−^ concentration using acid-base titration with 0.1 M H_2_SO_4_ and methyl orange indicator, whereas the raw solution samples were subjected to measurement of H^+^ concentration using 0.1 M NaOH and phenolphthalein as an indicator. This allowed for the quantitative evaluation of the concentrations of generated acid and recovered base. Additionally, Na^+^ concentration in each solution was accurately determined using inductively coupled plasma atomic emission spectrometry. Variations in solution volume before and after the experiment were measured using the graduation marks on each container, and the water migration rate was subsequently calculated. Furthermore, the stored current and electrical conductivity data recorded during the experiment were used to calculate the ion flux, energy consumption, and current efficiency. The experimental data were analyzed to calculate the base recovery rate (Recovery, %), current efficiency (Current efficiency, %, CE), water migration rate (Water migration, W mig (%)), energy consumption per kilogram of Na_2_SO_4_ processed (Energy consumption, kWh/kg Na_2_SO_4_, E_1_), energy consumption per kilogram of NaOH produced (Energy consumption, kWh/kg NaOH, E_2_), and ion flux (Ion flux, mol/m^2^·h, J).

The base recovery rate was calculated using the following Equation (1):

(1) $$Recovery\;\left(\%\right)=\frac{m_{f}}{m_{i}}\times 100$$

where $m_{f}$ represents the amount of Na^+^ (in moles) increased in the base chamber, and $m_{i}$ denotes the initial amount of Na^+^ (in moles) present in the feed solution.

Current efficiency was calculated using Equation (2) as follows:

(2) $$CE\;\left(\%\right)=\frac{{∆n}_{NaOH}}{I\cdot t/(z\cdot F)}\times 100$$

where ${∆n}_{NaOH}$ is the experimentally measured increase in the number of moles of NaOH in the base chamber, I is the applied current (A), t is the operation time (s), z is the ionic charge number (for Na^+^, z = 1), and F is Faraday’s constant (96,485 C·mol^−1^).

Water migration was calculated using Equation (3) as follows:

(3) $$W_{mig.}\left(\%\right)=\frac{V_{f}-V_{i}}{V_{i}}\times 100$$

where $V_{i}$ and $V_{f}$ represent the initial volume of the solution and the volume after the experiment, respectively.

Energy consumption ${(E}_{1})$ was calculated based on the Na_2_SO_4_ throughput (kg) using Equation (4) as follows:

(4) $$E_{1}=\frac{I\times \int_{0}^{t}Udt}{W}$$

where U is the applied voltage (V), I is the applied current (A), t is the required time (h), and W is the initial weight of Na_2_SO_4_ (kg).

Energy consumption ${(E}_{2})$ calculated based on the NaOH production (kg) using Equation (5) as follows:

(5) $$E_{2}=\frac{U\times I\times t}{m_{NaOH}}$$

where U is the applied voltage (V), I is the applied current (A),t is the required time (hr), and $m_{NaOH}$ is mass of produced NaOH (kg).

The ion flux (mol/m^2^h, J) was calculated using Equation (6) as follows:

(6) $$J=\frac{m_{f}}{At}$$

where $m_{f}$ is the number of moles of Na^+^ that moved to the base recovery chamber, A is the effective membrane area (m^2^) of the ion exchange membrane, and t is the time (h) required for the process.

## 3. Results

### 3.1. Effect of Current Density

**Figure 3:**
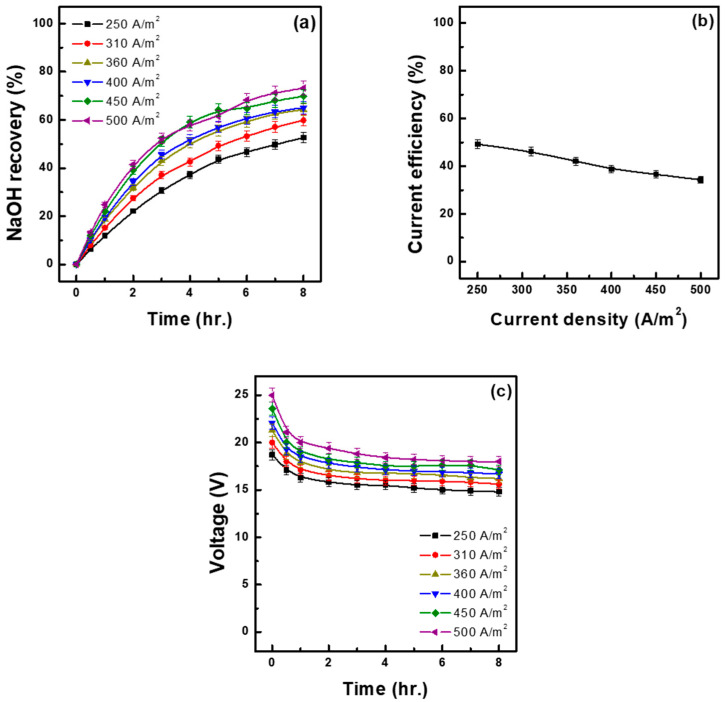
NaOH recovery at different current densities over time (**a**), relationship between current density and current efficiency (**b**) and cell voltage–time profiles at different current densities (**c**). (initial feed sol.: 1.30 M Na_2_SO_4_ 1.5 L; initial base sol.: 0.1 M NaOH 1.5 L, 30 °C).

To investigate the effect of current density on base recovery rate and current efficiency under operation at constant-current conditions, current density during the experiments was adjusted to 250, 310, 360, 400, 450, and 500 A/m^2^. The experiments were terminated when the experimental time reached 480 min. The raw material solution tank was filled with 1.5 L of 1.30 M Na_2_SO_4_ solution, whereas the base recovery tank was filled with 1.5 L of 0.1 M NaOH solution. The results of the experiments are presented in Figure 3 and Table 3 In the figure, Figure 3a shows the base recovery rate as a function of time, and Figure 3b shows current efficiency at each current density. As current density increased, the recovery rate increased, from a minimum of 52.60% at 250 A/m^2^ to a maximum of 73.19% at 500 A/m^2^. In contrast, current efficiency showed the opposite trend, decreasing with increasing current density, from a peak of 49.26% at 250 A/m^2^ and to 34.27% at 500 A/m^2^. This phenomenon is due to the fact that, as current density increases, ions accelerate, leading to the accumulation of large amounts of NaOH, which causes reverse diffusion of Na^+^ through the CEM in the opposite direction. Additionally, as ion mobility increases, the concentration of Na^+^ in the feed solution decreases, reducing the number of ions that can pass through the CEM. This leads to an increase in membrane resistance and overall stack resistance, requiring more energy for residual Na^+^ to pass through the membranes. This dynamic explains the observed decrease in current efficiency with increasing current density, which has been similarly reported in Lu et al. [21]. Higher current densities corresponded to higher cell voltages (Figure 3c), consistent with the increased resistance that contributed to reduced current efficiency and altered ion flux. Table 3 shows the water transfer rate, base recovery rate and recovery concentration, acid concentration in the recovered composite salt, ion flux, and energy consumption. The water transfer rate increased with increasing current density, reaching 17.55% at 500 A/m^2^, which was twice as high as the water transfer rate at 250 A/m^2^. In the two-liquid-phase system with CEMs and BPMs, the H^+^ and SO_4_^2−^ generated by each BPM in the feed solution chamber combine to form H_2_SO_4_, which then combines with residual Na_2_SO_4_ to form a composite salt. In this study, the highest acid concentration in this complex salt (1.19 M) was observed at 500 A/m^2^, the current density at which the base recovery rate was the highest. Ion mobility increased with current density, reaching a maximum of 6.51 mol/m^2^h at 500 A/m^2^. Energy consumption was lowest (1.30 kWh/kg) at 250 A/m^2^, increasing with increasing current density up to 2.31 kWh/kg at 500 A/m^2^. Increasing current density is advantageous for improving the base recovery rate and recovery concentration, but it has drawbacks, such as reduced current efficiency, increased energy consumption, and increased initial applied voltage. In particular, an excessive initial voltage can negatively affect the membrane’s lifespan; therefore, excessively increasing current density is not recommended. Conversely, a lower current density is advantageous in terms of current efficiency and energy consumption, but it significantly reduces the base recovery rate and recovery concentration, meaning that an excessively low current density would also be inappropriate. Overall, the suitable level should be determined by comprehensively considering both current efficiency and recovery rate. From this perspective, a current density of 450 A/m^2^ is considered the most appropriate in terms of balancing these two factors.

### 3.2. Effect of the Initial Feed Solution Concentration

**Figure 4:**
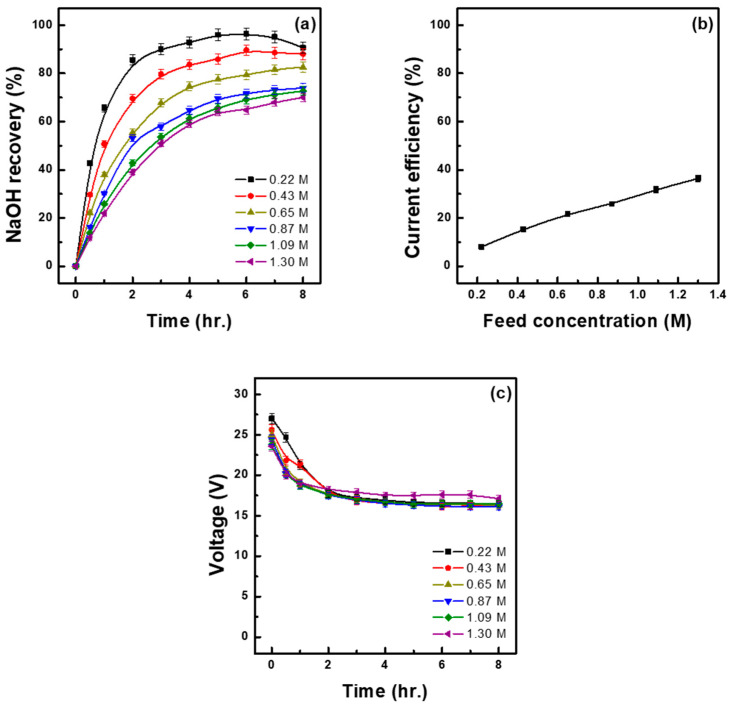
NaOH recovery at different Na_2_SO_4_ concentrations of the initial feed solution (M) over time (**a**), relationship between current efficiency and concentration of the feed solution (**b**) and cell voltage–time profiles at different current densities (**c**). (initial base sol.: 0.1 M NaOH 1.5 L, 30 °C).

To investigate the effect of the concentration of the initial feed solution on the base recovery rate and recovery concentration, experiments were conducted by adjusting the concentration of the raw material solution (Na_2_SO_4_) to 0.22, 0.43, 0.65, 0.87, 1.09, and 1.30 M under a current density of 450 A/m^2^. The results are presented in Figure 4 and Table 4. The applied Na_2_SO_4_ concentration of 0.22 M achieved the highest base recovery rate (90.64%) but the lowest recovery concentration (0.42 M) (Figure 4a and Table 4). On the other hand, 1.30 M Na_2_SO_4_ resulted in the lowest base recovery rate (70.00%) but the highest recovery concentration (1.67 M). This inverse relationship between recovery rate and recovery concentration is due to the total amount of ion transfer being limited under constant-current conditions. Under low-concentration conditions, the ratio of recovered Na^+^ to supplied Na^+^ is relatively high, resulting in a high recovery rate. In contrast, under high-concentration conditions, despite an increase in the total number of supplied ions, the number of ions that can move due to the limited current remains constant, leading to a decrease in the recovery rate. On the other hand, in terms of base recovery concentration, ion transfer efficiency decreases at low concentrations of the feed solution due to the relatively low supply of Na^+^. However, at high concentrations, the increased supply of Na^+^ results in higher transport efficiency, mobilizing more Na^+^ under the same current conditions and thus recovering a relatively higher base concentration. This is supported by the fact that the Na^+^ flux was lowest at 0.22 M Na_2_SO_4_ (1.34 mol/m^2^h) and increased continuously as the concentration of the feed solution increased, reaching the highest value at 1.30 M Na_2_SO_4_ (6.17 mol/m^2^h). Similar results were reported by Li et al. [22] Figure 4b shows the current efficiency at each tested concentration of the feed solution, with values increasing as the concentration increased, the lowest and highest being observed at 0.22 M Na_2_SO_4_ (7.92%) and 1.30 M Na_2_SO_4_ (36.39%), respectively. This trend is due to the fact that in high-concentration solutions of Na_2_SO_4_, the supply of Na^+^ is sufficient, so the current is used less for side reactions (such as water electrolysis) and more for Na^+^ transport. In contrast, in low-concentration solutions of Na_2_SO_4_, the supply of Na^+^ is insufficient, and some current is thus consumed in side reactions, leading to a decrease in current efficiency. Higher Na_2_SO_4_ concentrations resulted in lower cell voltages (Figure 4c), reflecting reduced resistance, which supports the improved current efficiency and Na^+^ flux observed under these conditions. As shown in Table 4, the acid concentration in the composite salt also increased with the concentration of the raw material solution, as observed for the base recovery concentration, reaching the highest value of 1.12 M under 1.30 M Na_2_SO_4_. Energy consumption showed the opposite trend, increasing as the concentration of the feed solution decreased. At 0.22 M Na_2_SO_4_, energy consumption was 8.56 kWh/kg, approximately 4.6 times higher than that recorded at 1.30 M Na_2_SO_4_ (1.86 kWh/kg). Feng et al. conducted experiments by varying the concentration of the raw material solution (Na_2_SO_4_) under a constant voltage of 12 V. The results showed that as the Na_2_SO_4_ concentration increased from 10 g/L to 30 g/L, energy consumption decreased from 1.23 kWh/kg to 0.82 kWh/kg [23]. Given that higher concentrations of Na_2_SO_4_ in the raw material solution result in lower energy consumption and higher current efficiency, using such highly concentrated solutions is advantageous from an economic perspective. Additionally, this approach is considered effective because it allows for the recovery of higher base concentrations.

### 3.3. Effect of Initial Base Concentration

**Figure 5:**
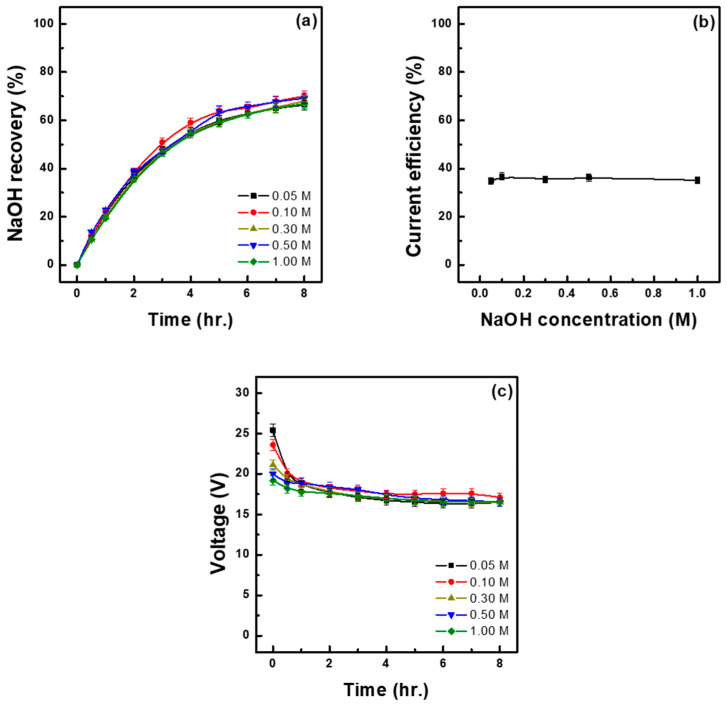
NaOH recovery at different initial NaOH concentrations (M) over time (**a**), relationship between initial NaOH concentration and current efficiency (**b**) and cell voltage–time profiles at different current densities (**c**). (initial feed sol.: 1.30 M Na_2_SO_4_ 1.5 L, 30 °C).

To determine the effect of initial base concentration, experiments were conducted by varying the concentration of NaOH fed into the base recovery chamber, specifically by adjusting it to 0.05, 0.10, 0.30, 0.50, and 1.00 M. Current density was set at 450 A/m^2^, and 1.5 L of 1.30 M Na_2_SO_4_ solution was loaded into the feed solution chamber. The base recovery chamber was filled with 1.5 L of NaOH solution at each of the above-mentioned concentrations. As shown in Figure 5a and Table 5, the highest (70.00%) and second highest (69.21%) base recovery rates were observed at initial NaOH concentrations of 0.10 M and 0.50 M, respectively. On the other hand, the lowest recovery rate (66.39%) was achieved at 0.05 M NaOH, although the difference between highest and lowest values was not significant. Figure 5b shows the current efficiency under each tested NaOH concentration, revealing the highest (36.70%) and lowest (34.82%) values at 0.10 M and 0.05 M NaOH, respectively, though the difference between them was not significant. This was due to the fact that under constant-current conditions, the amount of Na^+^ moving per unit time is nearly constant, and the base recovery chamber only accepts Na^+^ that has moved through the CEM from the feed solution; therefore, the initial base concentration is believed to influence the system’s ion transport environment or membrane concentration gradients rather than directly affecting the base recovery rate or current efficiency. On the other hand, the initial NaOH concentration directly influences the final concentration of the recovered base. According to the results shown in Table 5, the initial NaOH concentration of 0.05 M resulted in the lowest recovery concentration (1.59 M), whereas at 1.00 M NaOH, the recovered base reached a concentration of 2.26 M, increasing by approximately 0.7 M. Wei et al. conducted experiments by varying the initial NaOH concentration from 0.00 to 0.25 M in a two- compartment structure using CEMs and BPMs. The results showed that when an excessively low initial NaOH concentration or pure water was applied, initial voltage significantly dropped, leading to increased internal resistance and, consequently, higher energy consumption. Additionally, they observed that the amount of generated NaOH decreased as the initial NaOH concentration increased, and attributed this trend to the back-diffusion of OH^-^ and the inhibition of water dissociation reactions within the BPM due to increased osmotic pressure [24]. Similar trends were observed in this study, with current efficiency being the lowest at 34.82% and energy consumption being the highest at 1.87 kWh/kg under the initial NaOH concentration of 0.05 M. Excessively low initial base concentrations led to higher cell voltages (Figure 5c), consistent with the increased resistance that limited Na^+^ transport and lowered current efficiency. On the other hand, under 1.00 M NaOH, the increase in recovery concentration was smaller than the increases observed under the other NaOH concentrations, and current efficiency at 1.00 M NaOH was also lower than the values detected under 0.10–0.50 M. These results suggest that an excessively low initial base concentration may lead to reduced energy efficiency due to increased electrochemical resistance, whereas an excessively high initial concentration may limit the increase in the concentration of the recovered base. Therefore, an initial base concentration ranging between 0.1 and 0.5 M is considered optimal to ensure both energy efficiency and process stability.

### 3.4. Effect of Initial Base Volume

**Figure 6:**
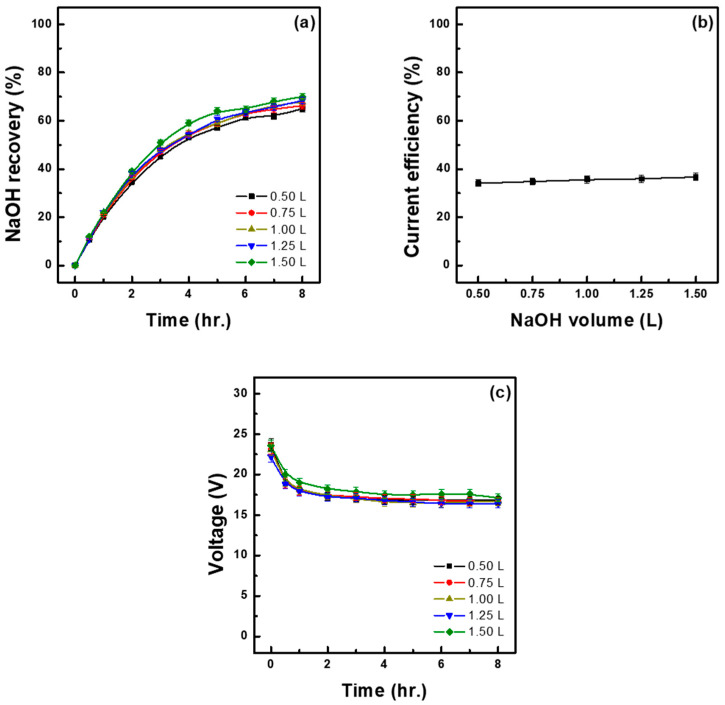
NaOH recovery under different initial NaOH solution volumes (L) over time (**a**), relationship between initial NaOH solution volume (L) and current efficiency (**b**) and cell voltage–time profiles at different current densities (**c**). (initial feed sol.: 1.30 M Na_2_SO_4_ 1.5 L, initial base: 0.1 M NaOH, 30 °C).

To increase the concentration of recovered NaOH, experiments were conducted by adding varying initial volumes of 0.1 M NaOH solution (range 0.50–1.50 L) to 1.5 L of 1.30 M Na_2_SO_4_ raw material solution. As shown in Figure 6a, the base recovery rate was highest (70.00%) under the application of the largest initial volume of NaOH solution (1.5 L) and progressively decreased as the volume decreased, reaching the lowest value of 65.07% at 0.50 L. This declining trend is thought to be primarily due to the increased electrical resistance of the ion exchange membrane caused by the high NaOH concentration, coupled with the promoted back-diffusion of anions such as SO_4_^2−^. More specifically, when the initial NaOH solution volume is small, even if the same amount of Na^+^ passes through the membrane, the NaOH concentration in the recovery chamber sharply increases, leading to a large ion concentration gradient between the two sides of the membrane and to concentration polarization due to the potential gradient. This in turn increases the membrane’s internal electrical resistance, thereby reducing the migration rate of Na^+^ and causing the back-diffusion of anions such as SO_4_^2−^, which decreases current efficiency. Such current loss leads to a reduction in the amount of Na^+^ transported, which results in a decrease in the molar amount of recovered NaOH as well as in an overall reduction in the base recovery rate. As shown in Table 6, the highest (36.70%) and lowest (34.12%) current efficiencies were observed at initial NaOH solution volumes of 1.50 L and 0.50 L, respectively. Therefore, the decrease in base recovery rate was directly related to the reduction in current efficiency. This trend has also been reported in Gonzalez et al., where it was observed that when a high-concentration LiOH solution comes into contact with an ion exchange membrane, concentration polarization intensifies due to an increase in the concentration gradient at the membrane surface, resulting in a decrease in lithium ion transfer efficiency. In particular, under high-concentration conditions such as 8.0 wt.% LiOH at a current density of 1100 A/m^2^, the number of transferred ions decreased by up to 64% [25]. In contrast, the concentration of recovered NaOH exhibited the opposite trend. That is, the smaller the initial NaOH solution volume, the more Na^+^ accumulated in a smaller volume of the recovery solution, resulting in a relatively higher concentration of recovered NaOH. According to our experimental results, the highest (2.85 M) and lowest (1.67 M) recovery concentrations were observed when using 0.50 L and 1.50 L of NaOH solution, respectively. As shown in Table 6, the water transfer rate increased as the initial NaOH solution volume decreased, peaking at 76.82% under the smallest tested volume of 0.50 L. Such rate was higher than those obtained with the other tested volumes, even considering the initial difference in volume. In fact, under 0.50 L of NaOH solution, the volume increased by 0.38 L, whereas under 1.50 L, it increased by 0.24 L. Due to this water transfer rate, the concentration of recovered NaOH began to decrease after 420 min when 0.50 L of NaOH solution was applied, as similarly reported in Cho [26] and Lee et al. [19]. Notably, Lee et al. reported that setting the initial NaOH solution volume to 0.50 L under a constant voltage of 20 V resulted in a high water migration rate of 101.9%, which led to a decline in recovery concentration over time [19]. Energy consumption was highest at 1.91 kWh/kg under the application of 0.50 L of NaOH solution and lowest at 1.82 kWh/kg under the 1.00 M and 1.25 M conditions. The highest concentration of recovered NaOH (2.85 M) was achieved under 0.50 L of NaOH solution (the smallest initial volume tested), making this an optimal volume for obtaining high NaOH concentrations. However, such a small initial volume implies a high water transfer rate, increasing the likelihood of solution dilution as well as increasing energy consumption, which may negatively impact overall process efficiency. On the other hand, compared with 0.50 L of NaOH solution, the addition of 1.00 L resulted in a lower recovery concentration of 2.13 M but required the lowest energy consumption while maintaining a relatively high current efficiency, making it a more suitable initial volume to ensure long-term operational stability and energy efficiency.

### 3.5. Optimal Process Design

To derive the optimal operating conditions, the effects of current density, feed solution concentration, and the initial concentration and volume of the base solution were comprehensively examined under a constant-current two-compartment BMED system. Regarding current density, higher values favored increased NaOH recovery and concentration, whereas lower current densities exhibited better current efficiency and lower energy consumption. These results highlight a trade-off between recovery performance and energy efficiency, implying that current density should be optimized by balancing both parameters. Accordingly, a current density of 450 A/m^2^ was deemed most suitable. Increasing concentration of the feed solution increased the amount of Na_2_SO_4_ processed per batch, improved current efficiency, and reduced energy consumption. Therefore, 1.30 M Na_2_SO_4_ was identified as the most economically feasible condition. To enhance the concentration of the recovered base, the initial NaOH concentration and volume were adjusted. Notably, the optimal initial NaOH concentration range was 0.10–0.50 M. When the concentration was <0.10 M, energy efficiency declined because of increased electrochemical resistance. In contrast, >1.00 M concentrations exhibited diminishing returns regarding increased recovery concentration. The initial volume of NaOH solution also played a critical role, depending on the target application. When 0.50 L of NaOH solution was used, the recovered base reached the highest concentration of 2.85 M, making it suitable for applications requiring high-concentration alkali. However, this condition was accompanied by a higher water migration rate and increased energy consumption, reducing overall process efficiency. In contrast, using 1.00 L of NaOH resulted in a slightly lower recovery concentration of 2.13 M, provided the lowest energy consumption and relatively high current efficiency, indicating favorable long-term operational stability and energy efficiency.

Specifically, 2.85 M NaOH (approximately 9.96%) was obtained under conditions optimized for high concentration, whereas 2.13 M NaOH (approximately 7.75%) was recovered under conditions optimized for energy and process stability. Although these concentrations fall short of commercial-grade NaOH specifications, the recovered alkali can still be reused within the precipitation or solvent extraction stages of lithium-ion battery recycling processes. Mixing the recovered NaOH with supplementary commercial NaOH could reduce the overall chemical costs in such applications.

Meanwhile, sulfuric acid was recovered in the feed chamber as a complex salt mixture containing residual Na_2_SO_4_, limiting its direct applicability in pure form. To enable the recovery of high-purity sulfuric acid, further research into effective removal processes for residual Na_2_SO_4_ is required.

### 3.6. Treatment of Complex Salts

In this study, we successfully recovered high-purity NaOH from the base recovery chamber using a two-liquid BMED system equipped with CEMs and BPMs. However, a problem arose in the feed solution chamber due to the formation there of a complex salt composed of H_2_SO_4_ and Na_2_SO_4_. In such cases, to efficiently recover H_2_SO_4_ from this complex salt, it would be necessary to remove residual Na_2_SO_4_ using an appropriate removal technique. One method to achieve this is through anti-solvent crystallization, a process that involves adding methanol to induce selective precipitation. Methanol is an organic solvent with very low solubility in Na_2_SO_4_. When added to a Na_2_SO_4_ aqueous solution, it immediately modifies the solvent composition, leading to a sharp decrease in Na_2_SO_4_ solubility and causing the solution to reach a supersaturated state. Consequently, Na_2_SO_4_ precipitates as Na_2_SO_4_·10H_2_O as a result of the decrease in the solvent’s dielectric constant and the desolvation of ions caused by methanol addition, shifting the dissolution equilibrium toward precipitation. The formed hydrate can be easily removed by physical methods such as filtration, whereas H_2_SO_4_, which is highly soluble in methanol, remains in the solution, enabling selective removal of Na_2_SO_4_. In fact, Cho et al. observed the formation of precipitate during the ED process due to high concentrations of Na_2_SO_4_ and conducted experiments using methanol as a precipitant to remove this salt. The results showed that adding methanol at a volume ratio of 0.4 relative to the feed solution achieved Na_+_ and SO_4_^2−^ removal rates of approximately 65% and 51%, respectively [27]. Additionally, Huang et al. investigated the selective separation of Na_2_SO_4_ and sodium chloride (NaCl) from spent electrolyte solution using various organic solvents, including methanol and ethanol, as antisolvents. Specifically, methanol was added at a 1:1 (Vm:Vs) ratio to a saturated solution of Na_2_SO_4_ (195 g/L) and NaCl (318 g/L), followed by stirring for 2 h, centrifugation, filtration, and drying at 70 °C for 24 h. Subsequently, Na_2_SO_4_ was recovered with a yield of 92.85% and a purity of 92.41%, whereas NaCl obtained from the residual solution via evaporation crystallization exhibited a purity of 97.41% [28].

However, when methanol is mixed with sulfuric acid, the highly toxic compound dimethyl sulfate (DMS) may be generated, which is strongly corrosive, severely irritating to the respiratory tract, and classified as a probable human carcinogen [29,30]. In addition to these safety concerns, scaling up the proposed anti-solvent crystallization process requires additional steps for methanol recovery and recycling, which substantially increase energy demand, process complexity, and operating costs. Moreover, in the case of complex salts mixed with H_2_SO_4_, the solubility of Na_2_SO_4_ may increase under low pH conditions, and exothermic reactions may occur when acids and alcohols are mixed, thereby posing additional risks to process stability. Therefore, the proposed anti-solvent crystallization should be regarded as a promising approach for H_2_SO_4_ recovery, but further studies are essential to ensure safety in acidic environments and to validate its efficiency and practical applicability.

## 4. Conclusions

This study investigated the recovery of caustic soda under constant-current conditions by applying a two-compartment bipolar membrane electrodialysis process to wastewater containing sodium sulfate (Na_2_SO_4_). The following main conclusions were drawn:

- As current density increased from 250 A/m^2^ to 500 A/m^2^, the base recovery rate showed an overall increasing trend, improving significantly by approximately 21%. However, excessive current density was shown to potentially lead to adverse effects such as reduced current efficiency, increased energy consumption, and increased initial applied voltage. Conversely, when current density was too low, limitations such as a reduced base recovery rate and recovery concentration were observed.
- As the concentration of the raw material solution increased, the base recovery rate decreased, whereas the recovery concentration actually increased. More specifically, 1.30 M Na_2_SO_4_ resulted in the lowest recovery rate of 70.00% but in the highest recovery concentration of 1.67 M. On the other hand, as the concentration of the raw material solution decreased, energy consumption increased, and current efficiency decreased. Therefore, from an economic perspective, it is more advantageous to use raw material solutions with higher concentrations.
- The initial concentration of the base does not directly affect the base recovery rate. However, if it is excessively low, it may lead to increased electrochemical resistance and reduced energy efficiency. Conversely, if it is excessively high, it may limit the increase in the concentration of the recovered base. Therefore, considering both energy efficiency and process stability, the optimal initial base concentration is within the range of 0.1–0.5 M.
- To increase the concentration of the recovered base, it is advisable to adjust the initial volume of the added base solution. The highest recovery concentration (2.85 M) was achieved when the initial volume was adjusted to 0.50 L, which is thus considered as the most suitable when the objective is to recover high-concentration bases. On the other hand, when the initial volume of the base solution was 1.00 L, the recovery concentration was 2.13 M (slightly lower than that achieved at 0.50 L) but resulted in the lowest energy consumption and a higher current efficiency, making this volume suitable for ensuring stability and efficiency in long-term operations.

## Figures and Tables

**Table 1:** Specifications of the bipolar membrane electrodialysis system used in this study.

**Effective membrane area**	0.055 m^2^ (11 ea BM/10 ea CEM)
**Operating mode**	Constant voltage/Constant current
**Terminating mode**	Current, Conductivity, Time, pH
**Terminating condition**	Current: 0.00–6.00 A Voltage: 0~30.0 V Conductivity: 0.0~300 mS/cm pH: 0~14 Time: 0–999 min
**Line capacity**	0.1 L
**Upper temperature limit**	40 °C
**Upper tank capacity limit**	2.5 L

**Table 2:** Detailed specifications of the bipolar and cation exchange membranes used in this study.

Membrane	BPM	CEM
**Type**	-	Strong acid (Na type)
**Characteristics**	Water splitting -Voltage 1.0 V -Efficiency ≥ 0.98	High mechanical strength /Alkali resistance
**Electric resistance (Ω** **·cm^2^)**	-	4.5
**Burst strength**	≥0.70	≥0.40
**Thickness (mm)**	0.28	0.21
**Temperature (** **°C)**	≤40	≤60
**pH**	-	0~14

**Table 3:** Effect of current density on time and water migration, NaOH recovery, acid in mixed solution, ion flux, and energy consumption (initial feed sol.: 1.30 M Na_2_SO_4_ 1.5 L; initial base sol.: 0.1 M NaOH 1.5 L, 30 °C).

Current Density (A/m^2^)	Time (h)	Water Migration to Base (%)	Recovery of NaOH (%) (M)	Acid in Mixed Sol. (M)	Ion Flux (mol/m^2^h)	Energy Consumption	
						(kWh/kg Na_2_SO_4_) *	(kWh/kg NaOH) **
250	8.00	8.69	52.60 (1.35)	0.80	4.68	1.30	4.39
310	8.00	10.17	59.84 (1.51)	0.88	5.31	1.49	4.42
360	8.00	12.50	64.29 (1.58)	0.97	5.72	1.68	4.64
400	8.00	14.24	65.05 (1.61)	1.03	5.88	1.86	5.07
450	8.00	15.73	69.82 (1.67)	1.12	6.21	2.05	5.21
500	8.00	17.55	73.19 (1.71)	1.19	6.51	2.31	5.60

* Energy consumption to process 1 kg of Na_2_SO_4_. ** Energy consumption to process 1 kg of NaOH.

**Table 4:** Effect of Na_2_SO_4_ concentration (M) of the initial feed solution on time and water migration, NaOH recovery, acid in mixed solution, ion flux, and energy consumption (initial base sol.: 0.1 M NaOH 1.5 L, 30 °C).

Initial Feed Sol. (M)	Time (h)	Water Migration to Base (%)	Recovery of NaOH (%) (M)	Acid in Mixed Sol. (M)	Ion Flux (mol/m^2^h)	Energy Consumption	
						(kWh/kg Na_2_SO_4_) *	(kWh/kg NaOH) **
0.22	8.00	16.45	90.64 (0.42)	0.25	1.34	8.56	16.77
0.43	8.00	17.13	87.98 (0.73)	0.47	2.61	4.31	8.70
0.65	8.00	17.25	82.61 (1.00)	0.64	3.67	3.03	6.51
0.87	8.00	14.19	73.91 (1.22)	0.78	4.38	2.51	6.03
1.09	8.00	15.53	72.73 (1.46)	0.96	5.39	2.05	5.00
1.30	8.00	15.68	70.00 (1.67)	1.12	6.17	1.86	4.72

* Energy consumption to process 1 kg of Na_2_SO_4_. ** Energy consumption to process 1 kg of NaOH.

**Table 5:** Effect of the NaOH concentration (M) of the initial base solution on time and water migration, NaOH recovery, acid in mixed solution, ion flux, and energy consumption (initial feed sol.: 1.30 M Na_2_SO_4_ 1.5 L, 30 °C).

Initial Base Sol. (M)	Time (h)	Water Migration to Base (%)	Recovery of NaOH (%) (M)	Acid in Mixed Sol. (M)	Ion Flux (mol/m^2^h)	Energy Consumption	
						(kWh/kg Na_2_SO_4_) *	(kWh/kg NaOH) **
0.05	8.00	11.76	66.39 (1.59)	1.02	5.90	1.87	5.00
0.10	8.00	15.68	70.00 (1.67)	1.12	6.23	1.86	4.72
0.30	8.00	14.65	67.99 (1.80)	1.06	6.06	1.83	4.78
0.50	8.00	17.13	69.21 (1.90)	1.09	6.15	1.83	4.70
1.00	8.00	20.30	67.00 (2.26)	1.08	5.96	1.84	4.87

* Energy consumption to process 1 kg of Na_2_SO_4_. ** Energy consumption to process 1 kg of NaOH.

**Table 6:** Effect of initial NaOH solution volume (L) on time and water migration, NaOH recovery, acid in mixed solution, ion flux, and energy consumption (initial feed sol.: 1.30 M Na_2_SO_4_ 1.5 L, initial base: 0.1 M NaOH, 30 °C).

Initial Base Sol. (L)	Time (h)	Water Migration (%)		Recovery of NaOH (%) (M)	Acid in Mixed Sol. (M)	Ion Flux (mol/m^2^h)	Energy Consumption	
		Base	Feed				(kWh/kg Na_2_SO_4_) *	(kWh/kg NaOH) **
0.50	8.00	76.82	−25.61	65.07 (2.85)	1.15	5.79	1.91	5.21
0.75	8.00	44.47	−22.23	66.32 (2.46)	1.14	5.89	1.88	5.03
1.00	8.00	28.80	−19.20	67.85 (2.13)	1.12	6.03	1.82	4.76
1.25	8.00	20.30	−16.92	68.41 (1.88)	1.11	6.08	1.82	4.72
1.50	8.00	15.68	−15.68	70.00 (1.67)	1.12	6.23	1.86	4.72

* Energy consumption to process 1 kg of Na_2_SO_4_. ** Energy consumption to process 1 kg of NaOH.

## Data Availability

All data generated or analyzed during this study are included in this published article.
